# 3D Bioprinting of Blood Vessel Model for Improving Wound Healing

**DOI:** 10.3390/ijms27094019

**Published:** 2026-04-30

**Authors:** Florin Iordache, Madalina Dulceanu, Alina Maria Holban, Alexandra Valentina Badaluta, Aurelia Magdalena Pisoschi, Bogdan Stefan Vasile, Bogdan Amuzescu, Carmen Curutiu

**Affiliations:** 1 Faculty of Veterinary Medicine, University of Agronomic Sciences and Veterinary Medicine of Bucharest, Splaiul Independentei 105, 050097 Bucharest, Romania; floriniordache84@yahoo.com (F.I.); aureliamagdalenapisoschi@yahoo.ro (A.M.P.); 2 SC Personal Genetics SRL, Strada Frumoasă 4, 010987 Bucharest, Romania; dulceanu.madalina@gmail.com; 3 Department of Microbiology, Faculty of Biology, University of Bucharest, Splaiul Independentei 91–95, 050095 Bucharest, Romania; badaluta.valentina-alexandra@s.bio.unibuc.ro (A.V.B.); carmen.curutiu@bio.unibuc.ro (C.C.); 4 National Centre for Micro and Nanomaterials & National Centre for Food Safety, Faculty of Chemical Engineering and Biotechnologies, National University of Science and Technology Politehnica Bucharest, Splaiul Independentei 313, 060042 Bucharest, Romania; bogdan.vasile@upb.ro; 5 Department of Anatomy, Animal Physiology and Biophysics, Faculty of Biology, University of Bucharest, Splaiul Independentei 91–95, 050095 Bucharest, Romania; bogdan.amuzescu@bio.unibuc.ro

**Keywords:** 3D bioprinting, blood vessels model, stem cells, tissue engineering

## Abstract

Hydrogel-based stem cell therapy uses different stem cells and bioactive molecules for wound healing in the treatment of diabetes and chronic burn wounds by accelerating angiogenesis, collagen deposition, and inhibition of inflammatory responses. Artificial vessels have already been used for patients with cardiovascular diseases, but most of them are polymeric, which can cause thrombosis and restenosis. 3D bioprinting combines cells, growth factors, and biomaterials to create a setting in which cells grow and differentiate into native tissue-like structures. The current study aimed to create a model of blood vessels using collagen and hyaluronic acid hydrogel combined with endothelial and muscle progenitor cells derived from amniotic mesenchymal stem cells using 3D bioprinting. A computer-aided design (CAD) software was employed to create the 3D models of a blood vessel model and printed using a 3D bioprinter with two printheads: one with bioink encapsulating endothelial progenitor cells and the second with bioink encapsulating smooth muscle progenitor cells. The blood vessel constructs were characterized morphologically and structurally by Fourier Transform Infrared (FTIR) Spectroscopy, thermogravimetric analysis (TGA), Scanning Electron Microscopy (SEM), immunohistochemistry, water uptake, and enzymatic degradation. Viability, proliferation, oxidative stress, vascular endothelial growth factor (VEGF) and nitric oxide (NO) production were assessed to demonstrate the cytocompatibility of the blood vessel constructs. Our results showed that collagen–hyaluronic acid hydrogels embedded with stem cells can be used for vascular constructs, meeting the desired requirements of biocompatibility and accuracy in reproducing the model created in the CAD software v1.0.

## 1. Introduction

Blood vessels are tubular networks that carry blood, exchange nutrients, and eliminate waste. These networks include arteries, arterioles, veins, venules, and capillaries. Thick-walled arteries manage high pressure, thin-walled veins employ valves, and microscopic capillaries facilitate nutrition exchange. They are composed of three primary layers (tunica intima, media, and externa). Most blood arteries, with the exception of capillaries, have a three-layered structure encircling the lumen. A thin layer of endothelium that lessens friction corresponds to tunica intima; tunica media, comprising elastic fibers and smooth muscle, is regulated by the autonomic nervous system to govern circulation and blood pressure through vasoconstriction and vasodilation, and tunica externa is composed of collagen, a connective tissue rich in extracellular matrix proteins that provides protection and structural support [1,2,3].

Most chronic vascular diseases, such as peripheral artery disease, carotid artery disease, chronic venous insufficiency, and aortic aneurysms, create blood vessel dysfunction. Like major wounds, atherosclerosis of large vessels, diabetes, or capillary damage led to tissue hypoxia, in which the wound is under-oxygenated, fibroblasts underperform, collagen deposition drops, and infection risk rises. Also, in many chronic diseases, the endothelium becomes pro-inflammatory, pro-thrombotic, and less able to deliver oxygen efficiently. Some chronic conditions increase clotting tendency or microvascular plugging and impair angiogenesis, leading to weak granulation tissue, fragile wound bed, and delayed closure [4,5,6].

Although autologous vascular grafts are the gold standard for clinical use, they are not suitable for many patients due to vascular disease, amputations, or previous vascular tissue harvesting. Despite the clear clinical needs of vascular substitutes, currently available synthetic grafts (for example, Dacron and Teflon) have proven to be successful only in large caliber implants; small-diameter vascular substitutes (<6 mm) have generally unsatisfactory results, with major negative side effects, such as acute thrombosis, hyperplasia, and aneurysm [7,8]. Arterial failures are generally attributed to the relatively low blood flow rate in these small vessels, which increases the rate of interactions between cells and molecules in the blood and polymeric implants. Therefore, there are strict requirements regarding the biofunctionality of the small-diameter-designed grafts. In order to produce grafts with normal vascular functions, at least two requirements must be met: the existence of confluent endothelium, providing a non-thrombogenic interface, and the presence of smooth contractile muscle tissue that can withstand hemodynamic stress and physiological relaxation of the vessel [8,9]. Tissue engineering has emerged as a promising alternative approach for viable small-diameter arterial grafts. Various strategies, including decellularized tissue, synthetic polymeric matrices, self-assembling cell layers, and hydrogels, have been used to create living vascular structures and replace native blood vessels; these strategies have achieved different levels of success [9,10,11].

Hyaluronic acid promotes fibroblast migration and proliferation, increases the synthesis of collagen and elastin, encourages angiogenesis, and controls inflammation, which speeds up wound healing and tissue regeneration with less scarring [12,13]. Advanced, biocompatible polymers such as collagen–hyaluronic acid hydrogels are utilized to create artificial blood vessels and encourage angiogenesis. These hydrogels provide high water retention and structural stability for cell encapsulation by imitating the natural extracellular matrix (ECM) to enhance the vascular structure and cellular growth [14]. These scaffolds can support the growth of homogeneous endothelium, which is crucial for the functionality of engineered blood vessels. They are among the most promising types of biomaterials for angiogenesis and tissue repair since they may be tailored to allow for particular mechanical characteristics and degradation rates [15].

Amniotic fluid stem cells (AFSCs) are a heterogeneous population of mesenchymal cells of fetal origin of great importance in regenerative medicine. The advantages of using AFSCs derive from the fact that they are a biological material that remains after prenatal diagnostic tests, have a high proliferation rate, have a population doubling time that is high, have a normal karyotype, have long telomeres, do not form tumors, have immunomodulatory capacity, and are multipotent. This population is known to have low immunogenicity, so it is less likely to generate an immune response, thus having a high therapeutic potential [16,17]. Phenotypes found in the cell culture of amniotic fluid include epithelial-like and fibroblast-like cells. There are also approximately 0.1–0.5% stem cells defined by c-kit expression (CD117+) on the cell surface. The regenerative potential of amniotic fluid-derived mesenchymal stem cells derives from their ability to differentiate into several types of cells [18], such as endothelial [19], cardiac [20], neural, and bone cells [21]. AFSCs can also be reprogrammed without the use of gene transfer technology for the induction of pluripotent stem cells [20]. The literature suggests a strong correlation between AFSCs and the cardiovascular system. AFSCs have demonstrated the ability to form capillary-like networks when grown in hydrogels, and there is also evidence of paracrine signaling when AFSCs are indirectly co-cultivated with human cardiac cells, thereby modulating cardiac regeneration [19,22].

For small-diameter arterial grafts, a promising alternative approach is 3D bioprinting. Minimizing cell loss, promoting cell–cell interactions, an increase in mechanical properties, and biocompatibility of scaffolds are among the main advantages of bioprinting. In 3D bioprinting, the scaffold is printed layer by layer, creating structures similar to natural organs or tissues [23]. Bioprinting involves several steps: (i) selection of the biomaterial, (ii) design of the bioprinting model/scaffold using CAM software v1.0, (iii) selection of the proper bioprinting technique, and (iv) analysis of bioprinted constructs [24,25]. An ideal biomaterial must comply with several properties in order to permit remodeling of the scaffold post-implant, such as being biocompatible, non-immunogenic, non-toxic, and anti-thrombotic, with vasoactive properties. Physicochemical parameters such as geometry, surface properties, adhesion, pore size, degradation, and biocompatibility are key factors for scaffold design [25,26,27]. Biological materials, synthetic polymers, hydrogels, extracellular matrix, cell aggregates, and microcarrier structures can be considered for obtaining natural-like tissue structures [27].

The aim of this study was to create a model of a small-diameter 2-layer blood vessel using collagen and hyaluronic acid hydrogels combined with endothelial and muscle progenitor cells derived from mesenchymal stem cells isolated from amniotic fluid using 3D bioprinting.

## 2. Results

### 2.1. Characterization of Collagen–Hyaluronic Acid Hydrogels

**Figure 1:**
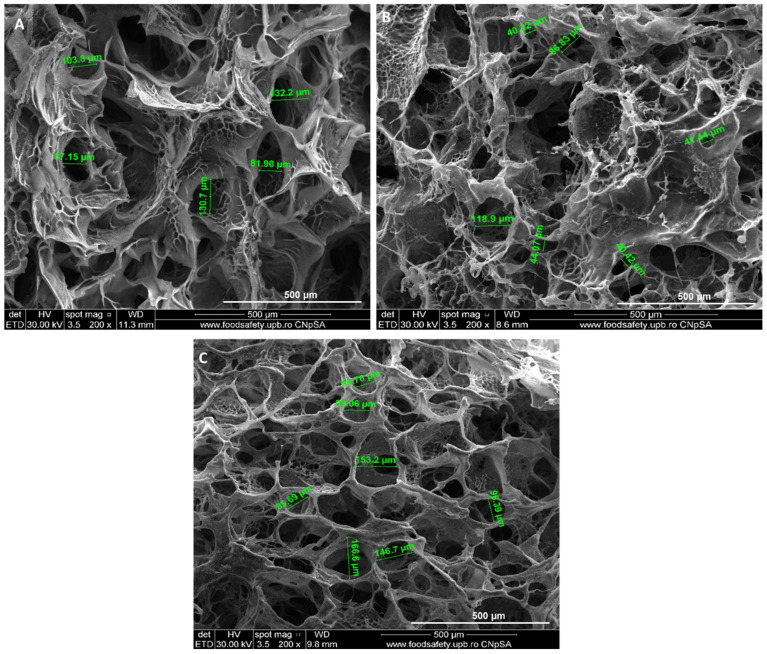
Scanning Electron Microscopy images representing microstructure of collagen–hyaluronic acid hydrogels with different ratios (**A**—H0 hydrogel; **B**—H2.5 hydrogel; **C**—H5 hydrogel). Green lines highlight pore sizes in the different samples.

**Figure 2:**
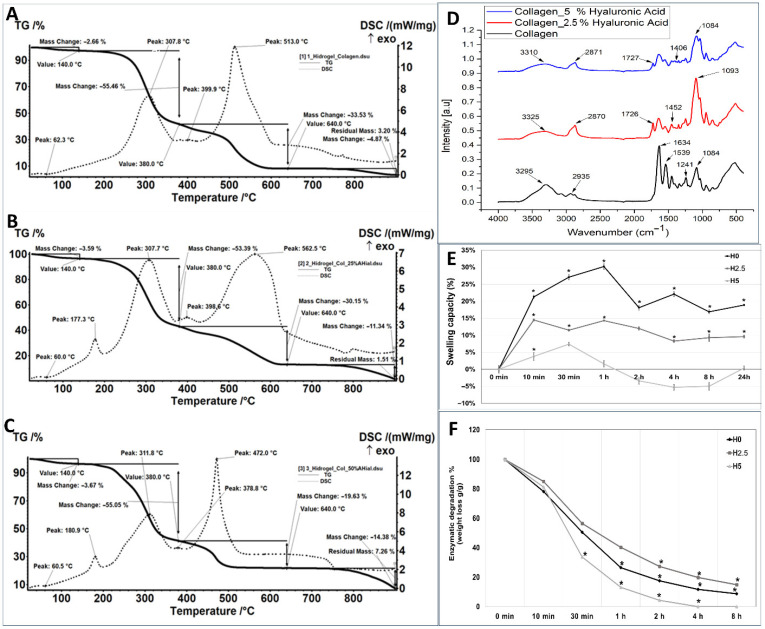
Thermogravimetric analysis (**A**–**C**), Fourier Transform Infrared Spectroscopy (**D**), swelling capacity (**E**), and enzymatic degradation (**F**) of collagen-hyaluronic acid hydrogels (H0; H2.5; H5), (*n* = 4, * *p* < 0.05, one-way ANOVA).

The morphology of collagen and collagen–hyaluronic acid hydrogels was assessed by SEM. The hydrogels present a characteristic porous morphology: the pore size was 107 µm ± 23 µm for H0 hydrogel (Figure 1A), 51 µm ± 20 µm for H2.5 hydrogel (Figure 1B), and 91 µm ± 7 µm for H5 hydrogel (Figure 1C), with a porosity between 63% and 70% (63% for H0, 64% for H2.5 and 70% for H5). Thermogravimetric analysis showed that the collagen hydrogel H0 loses 2.66% of its mass in the RT-140 °C range, the process being accompanied by an endothermic effect with a minimum at 62.3 °C. In the 140–380 °C range, the sample suffers a mass loss of 55.46%, the process being accompanied by a strong, broad exothermic effect, with a maximum at 307.8 °C. In the 380–440 °C range, a slower mass loss occurs, accompanied by a series of superimposed exothermic effects of lower intensity, indicating oxidation of some organic residues. Next, the carbon mass remaining after the oxidation undergoes a CO_2_ combustion process up to 640 °C. This burning process is accompanied by a very intense, slightly asymmetric exothermic effect with a maximum at 513 °C. The mass loss over the entire range is 33.53%. After 800 °C, the sample loses another 4.87% of its initial mass, with the residue representing 3.2% (Figure 2A). Hydrogels with 2.5% and 5% hyaluronic acid are like the collagen hydrogel, except for the exothermic effect at 177.3 °C and 180.9 °C, respectively, which could be due to a reaction that occurs between hyaluronic acid and collagen (Figure 2B,C). In the H2.5 and H5 hydrogels, the existence of a larger residue mass between 650 and 850 °C is observed as the percentage of hyaluronic acid increases, due to the sodium ions in sodium hyaluronate (Figure 2B,C).

The presence of hyaluronic acid in the hydrogels can be observed by comparing it with the spectrum obtained for the collagen hydrogel. The absorption band at approximately 3325 cm^−1^ (for the sample with 25% hyaluronic acid) and 3310 cm^−1^ (for the sample with 50% hyaluronic acid) is attributed to the OH and NH groups present in the structure of hyaluronic acid. At a wave number of approximately 2870 cm^−1^, the characteristic vibration of the C-H group is observed. The value of the wave number of approximately 1400 cm^−1^ is attributed to the COO- stretching characteristic of the acid group in hyaluronic acid. At approximately 1080 cm^−1^, the C-OH group is highlighted. The spectra recorded for the samples with hyaluronic acid indicate, at a wave number of approximately 1726 cm^−1^, the presence of carboxyl C=O groups (Figure 2D).

The water uptake was analyzed for 24 h. The H0 collagen hydrogel had the highest mass increase at 1 h with 30.2%, then started releasing some water at 2, 4, 8, and 24 h, reaching the values of 18.10%, 22.10%, 16.90%, and 18.90%, respectively, showing a stabilization of water uptake between 16 and 22% (Figure 2E). The hydrogels with 2.5% and 5% hyaluronic acid had a lower water uptake; the highest capacity was in the first hour, with the hydrogel mass increasing by 14.3% for H2.5 and 7.4% for H5. After one hour of immersion, the hydrogel mass did not increase by more than 10% for H2.5, and for H5, it did not change compared to the initial moment (Figure 2E). The biological stability of the hydrogels was assessed by the degree of degradation via collagenase (1 μg/mL) and hyaluronidase (10 μg/mL) because these enzymes can cleave hyaluronic acid and collagen completely. The enzymatic degradation of hydrogels was determined through weight loss, which is a function of the exposure time to collagenase and hyaluronidase solution. The fastest enzymatic degradation was observed in the H5 hydrogel: 2 h after incubation, only 4% remained, followed by H0 hydrogel, where after 2 h of enzymatic incubation, 18% of constructs remained. Interestingly, the slowest degradation was observed in H2.5, where the degradation period continued to 8 h (Figure 2F).

### 2.2. Characterization of Differentiated Endothelial Progenitor Cells (EPCs) and Smooth Muscle Progenitor Cells (SPCs)

**Figure 3:**
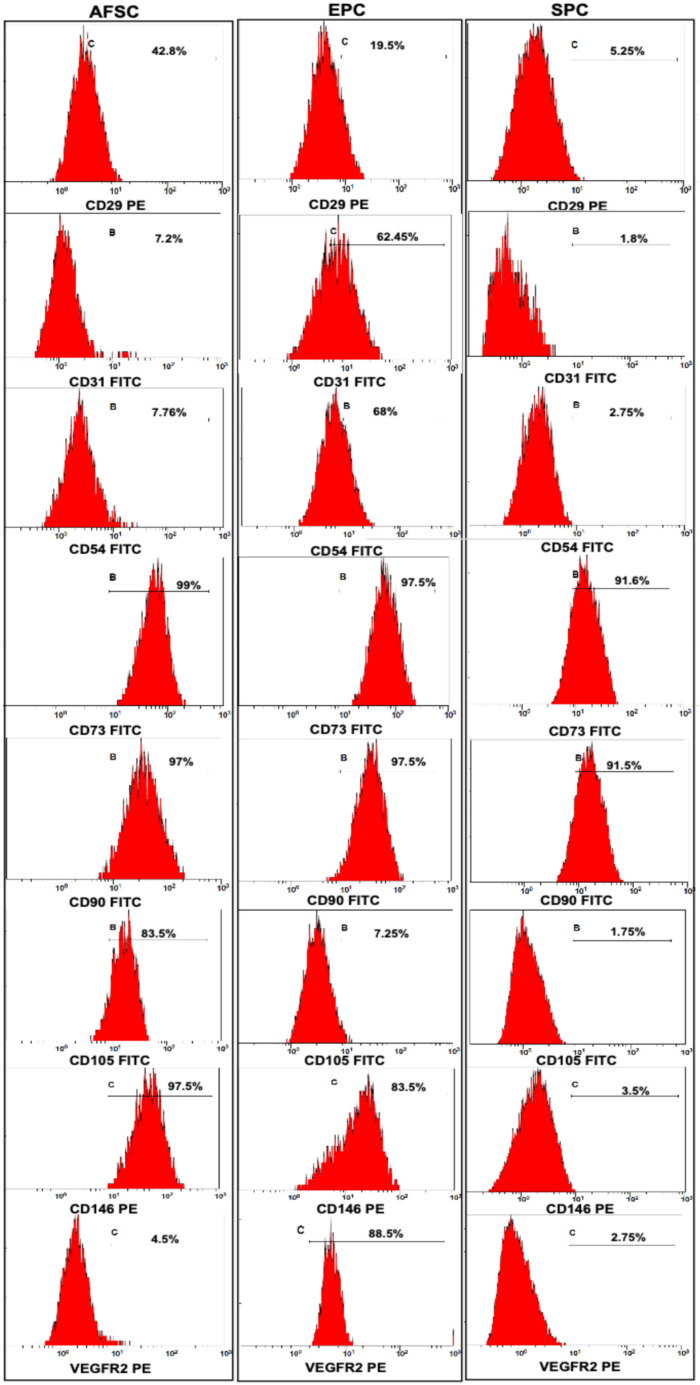
Flow cytometry assays for stem cell markers CD29, CD31, CD54, CD73, CD90, CD105, CD146, and VEGFR2 in AFSCs versus differentiated EPCs and SPCs cultivated in specific media supplemented with endothelial and muscular growth factors. Percentages of positive cells for each marker are indicated on the corresponding distribution histograms (*n* = 3, *p* < 0.05, one-way ANOVA).

Immunophenotyping the cells using flow cytometry with specific fluorescently labeled antibodies showed that the differentiation of AFSCs towards EPCs was achieved; the cells express specific endothelial markers such as CD29 (19.5%), CD31 (62.45%), CD54 (68%), CD105 (7.25%), CD146 (83.5%), and VEGFR2 (88.5%) (Figure 3). In SPCs, the expression of CD29 (5.25%), CD31 (1.8%), CD54 (2.75%), CD73 (91.6%), CD90 (91.5%), CD105 (1.75%), CD146 (3.5%), and VEGFR2 (2.75%) showed that the phenotype was changed compared to AFSCs, which highlights the following marker expressions: CD29 (42.8%), CD31 (7.2%), CD54 (7.76%), CD73 (99%), CD90 (97%), CD105 (83.5%), CD146 (97.5%) and VEGFR2 (4.5%) (Figure 3).

**Figure 4:**
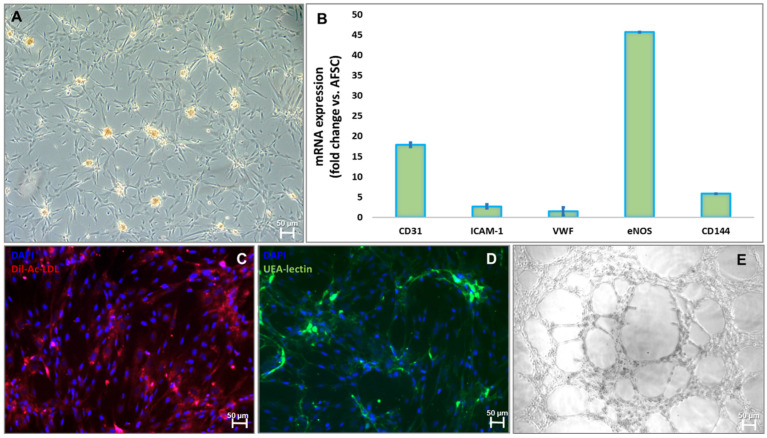
Markers for characterization of endothelial progenitor cell commitment: (**A**) Morphology of EPCs after 4 weeks of culture with endothelial growth factors (bFGF, IGF, VEGF, EGF); (**B**) mRNA expression levels for human CD31, ICAM-1, VWF, eNOS, CD144, (TaqMan qRT-PCR, *n* = 4, mean ± SEM, *p* < 0.05, one-way ANOVA); (**C**) Dil-Acetylated-LDL uptake (red); (**D**) UEA lectin (green); (**E**) tube-like structures of EPCs on a Matrigel extracellular matrix.

After 4 weeks of treatment with 10 ng/mL of bFGF, 40 ng/mL of VEGF, 10 ng/mL of EGF, and 20 ng/mL of IGF-1 in M200 medium, cultures of adherent AFSCs started to form colonies with epithelial-like “cobblestone” morphology (Figure 4A). Endothelial differentiation was confirmed by gene expression assay that showed increased mRNA expression for CD31 (18-fold), ICAM-1 (2.6-fold), VWF (1.5-fold), eNOS (45-fold), and CD144 (6-fold) (Figure 4B). Furthermore, functional characteristics of endothelial progenitor cells complete the molecular assay, displaying the binding capacity to acetylated-LDL (Figure 4C) and *Ulex europaeus* lectin (Figure 4D) tracers and the formation of a vascular-like structure when they are cultivated for 24 h on a Matrigel collagen matrix (Figure 4E).

**Figure 5:**
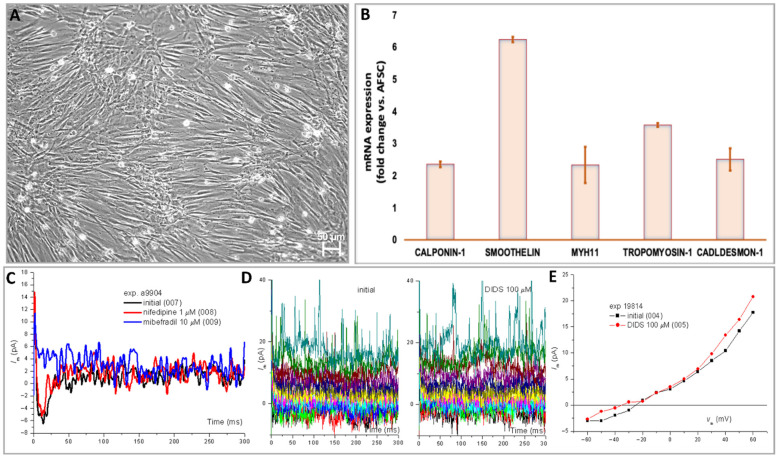
Markers for characterization of smooth muscle progenitor cell commitment: (**A**) Morphology of SPCs after 4 weeks of culture with specific growth factors (bFGF, EGF, heparin, IGF, BSA); (**B**) mRNA expression levels for human calponin-1, smoothelin, Mhy-11, TPM-1, and caldesmon-1 (TaqMan qRT-PCR, *n* = 4, mean ± SEM, *p* < 0.05, one-way ANOVA); (**C**) whole-cell patch-clamp assays in SPCs using double-ramp voltage-clamp protocol with traces recorded before and during application of nifedipine (1 µM) and mibefadril (10 µM) that blocked T-type Ca^2+^ current fluctuations measured in the depolarization step at −40 mV with the general IK protocol; (**D**) Effects of the Cl^−^ anion channel inhibitor DIDS (4,4′-diisothiocyano-2,2′-stilbenedisulfonic acid) on current traces recorded with the general IK protocol, and (**E**) modification of the I–V curve (points represent average current values during the 300 ms duration of the depolarization steps).

The treatment of AFSCs with 0.5 ng/mL of EGF, 2 ng/mL of bFGF, 5 ng/mL of heparin, 2 µg/mL of IGF, and 0.2 µg/mL of BSA in M231 medium induced differentiation toward smooth muscle cells, with some phenotypic changes such as spindle-shaped morphology and elongated actin-rich extensions (Figure 5A). The gene expression data showed upregulation of mRNA for calponin-1 (2.3-fold), smoothelin (6.2-fold), myh11 (2.3-fold), tropomyosin-1 (3.5-fold), and caldesmon-1 (2.5-fold) genes (Figure 5B). Whole-cell patch-clamp experiments performed on a total number of 24 SPCs, with applications of different pharmacological compounds, showed that nifedipine (1 µM) and mibefadril (10 µM) blocked T-type Ca^2+^ current fluctuations measured in the depolarization step at −40 mV with the general IK protocol (Figure 5C–E).

### 2.3. Biocompatibility and Immunohistochemistry of Blood Vessel Constructs

**Figure 6:**
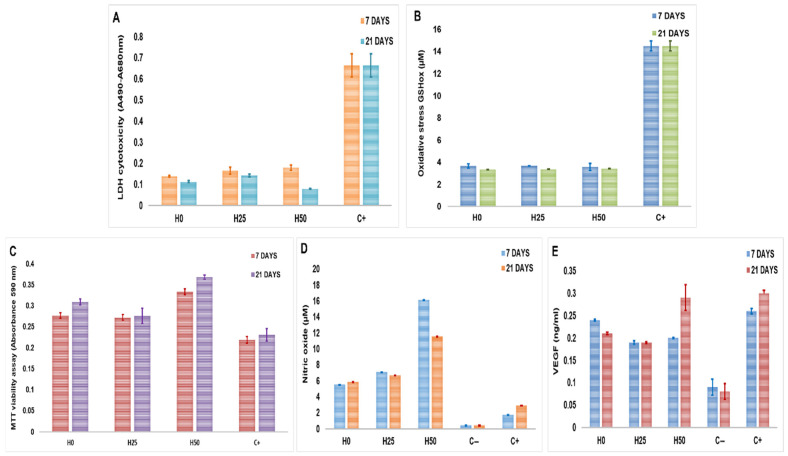
(**A**) LDH cytotoxicity assay. (**B**) GHS oxidative stress assay. (**C**) MTT proliferation assay. (**D**) Griess assay. (**E**) VEGF ELISA assay for bioprinted constructs at 7- and 21-day cultures (*n* = 4, *p* < 0.05, one-way ANOVA).

LDH (lactate dehydrogenase) viability assay measures the level of the enzyme in the cell culture media. LDH is a cytosolic enzyme; it is released into the cell culture media when plasma membrane damage occurs, indicating cellular death. At 7 days post-culture, the level of LDH was 0.138 (±0.0037) for H0 constructs, 0.165 (±0.015) for H2.5, and 0.180 (±0.0022) for H5 constructs compared with positive control from the assay (0.664 ± 0.054), suggesting that the hydrogels were not cytotoxic for the cells. At 21 days, the constructs H0 showed a value of 0.112 (±0.0054), and for H2.5 and H5 constructs, the values were 0.142 (±0.0056) and 0.0795 (±0.0022), respectively, with an insignificant decrease compared with the 7-day constructs (Figure 6A).

Furthermore, the oxidative stress was measured by GSH assay to quantify the level of oxidized glutathione. Glutathione is the highest concentration non-protein thiol in mammalian cells, between 0.5 and 10 mM. The concentration of oxidized glutathione was 3.65 µM (±0.20) in H0 constructs, 3.69 µM (±0.035) in H2.5, and 3.56 µM (±0.317) in H5 compared with the positive control of the assay that had a concentration of 14.47 µM (±0.0450) after 7 days in culture. After 21 days in culture, the concentration of oxidized glutathione was not significantly changed, with 3.33 µM (±0.011) for H0, 3.36 µM (±0.015) for H2.5, and 3.43 µM (±0.005) for H5 (Figure 6B). These data suggest that collagen–hyaluronic acid hydrogels do not create oxidative stress for EPCs and SPCs, similar to other studies that suggested that the normal level of oxidized glutathione falls between 10 and 50 µM, depending on the cell type [28,29].

The viability and proliferation of cells after 7- and 21-day cultures in the collagen–hyaluronic acid constructs were assessed by MTT assay, measuring the amount of formazan that results from converting the tetrazolium salts by mitochondrial enzymes. After 7 days, the proliferation rate increased with 26% in H0, 24% in H2.5, and 52% in H5 compared with control, represented by the cells cultured without hydrogels. After 21 days, the proliferation rate increased by 33% in H0, 19% in H2.5, and 59% in H5 compared with control (Figure 6C).

The nitric oxide secretion by EPCs was evaluated using the Griess assay. The concentration of nitric oxide was 5.5 µM (±0.0016) in H0 hydrogel, 7.08 µM (±0.005) in H2.5 hydrogel, and 16.15 (±0.0039) µM in H5 hydrogel compared with 1.17 µM (±0.004) in EPCs without hydrogel at 7 days post-cultivation. After 21 days, the nitric oxide concentration suffered minor modification, suggesting that the level of nitric oxide reached a plateau phase in this type of construct (Figure 6D).

The level of VEGF was measured using an ELISA assay; after 7 days, the concentration was 0.24 ng/mL in H0 constructs (±0.002), 0.19 ng/mL (±0.003) in H2.5, and 0.2 ng/mL (±0.001) in H5 constructs compared to 0.09 ng/mL (±0.018) in EPCs cultivated without hydrogel. The concentration of VEGF was increased after 21 days only for the H5 constructs (Figure 6E).

**Figure 7:**
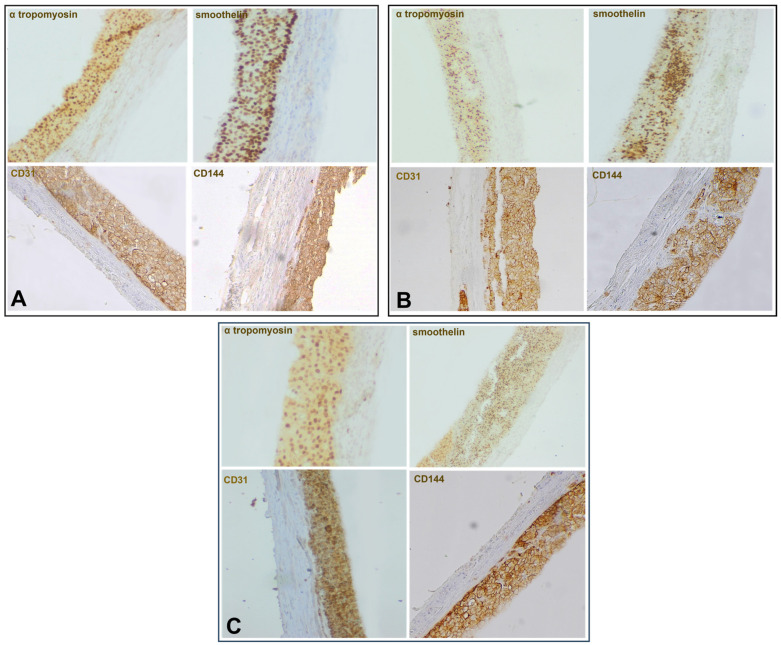
Immunohistochemistry of 21-day bioprinted constructs using monoclonal mouse anti-human CD31 (1:250), CD144 (5 μg/mL), smoothelin (1:100), and anti-human tropomyosin 1 (1:100). Visualization was achieved using a streptavidin–biotin peroxidase detection system with diaminobenzidine (DAB) as the chromogenic substrate (**A**—H0, **B**—H2.5, **C**—H5, Ob. 20×).

The constructs were cultivated in DMEM media supplemented with 10% FBS. The differentiation of EPCs and SPCs remains unchanged, as the immunohistochemistry markers showed after 21 days of culture. The immunohistochemistry showed that EPCs and SPCs remain in the hydrogel and continue the differentiation process towards endothelial and muscle cells. In the internal layer, CD31 (PECAM-1) and CD144 (VE-cadherin) expression for H0 constructs was 97% and 98%, respectively, for H2.5, 94% and 76%, and for H50, 100% and 97%. The endothelial progenitor cells create a uniform layer with strongly connected cells, adopting cobblestone morphology, suggesting that the collagen–hyaluronic hydrogels are optimal for proliferation and differentiation (Figure 7A–C). The external layers strongly stained for smoothelin and tropomyosin-1, with 99% for tropomyosin-1 and 96.4% for smoothelin in construct H0, 97% and 98% in H2.5, and 95.8% and 85% for H5, showing that collagen–hyaluronic acid hydrogels are suitable biomaterials for creating vascular structures that can promote neovascularization and wound healing (Figure 7A–C).

## 3. Discussion

In this study, a model of a small-diameter two-layer blood vessel using collagen and hyaluronic acid hydrogels combined with endothelial and muscle progenitor cells derived from mesenchymal stem cells isolated from amniotic fluid using 3D bioprinting was created. For vascular constructs, cell density and porosity are essential parameters. Cell density is crucial for determining the optimal printing strategy, influencing the viscosity of the bioinks. The higher the cell density, the higher the average viscosity, which can impact the fidelity of the printed tissue or organ [30]. Using 10 million cells per milliliter of bioink, we obtained vascular constructs with an internal diameter of 2.43 mm (±0.32), wall thickness of 1.45 mm (±0.14), and a height of 2.79 mm (±0.05). Fedorovich et al. showed that 5 to 10 million cells per milliliter of bioink may be ideal for bone tissue engineering [31,32]. The obtained hydrogels showed a morphology with an average pore size between 51 and 107 µm and a porosity between 63% and 70%. Other studies identified the ideal ranges for infiltration of particular cell types as pores with diameters of 200–750 μm for long peripheral axons, 200–400 μm for chondrogenic and osteogenic differentiation of mesenchymal stem cells, 50–160 μm for smooth muscle and endothelial cells, nerve cells, and fibroblasts, and <38 μm for microvascular epithelial cells [33,34]. Physicochemical determinations realized by TGA and FTIR give us important information about interactions of collagen–hyaluronic acid biomaterials, demonstrating that hyaluronic acid was incorporated into collagen hydrogels. Another important parameter for the collagen–hyaluronic acid hydrogels was represented by water uptake. The literature showed that polymer–polymer interactions and solutions with different pH can contribute to water uptake [35]. The results showed that collagen hydrogel had the highest water uptake after 1 h with 30.2%, followed by hydrogels with 2.5% and 5% hyaluronic acid, with a mass increase of 14.3% and 7.4%, respectively. Although all our samples permit water uptake, the simple collagen hydrogel presents the higher water uptake compared with the hydrogels that contain hyaluronic acid, probably due to the water molecules that are already present in its structure. Hyaluronic acid–collagen hydrogels expand when they are submerged in solutions with different osmolarities because the hyaluronic acid absorbs water, which causes the collagen network to stretch and become stressed. In this manner, mixed gels with residual stress, such as collagen fibers in tension and hyaluronic acid in compression, can be created, as presented by Lai et al. [36]. Regarding enzymatic degradation, the results showed that the fastest enzymatic degradation was observed in the H5 hydrogel, followed by the H0 hydrogel. Interestingly, the slowest degradation was observed in the H2.5 hydrogel, where the degradation period continued at 8 h. A possible explanation for the fastest degradation observed in the H5 hydrogel could be precisely the amount of hyaluronic acid, a molecule that acts like a sponge that retains water and facilitates the action of enzymes. The rapid degradation rate may also be attributed to the collagen hydrogel, whose composition was constituted from short-chain collagen peptides, not from long collagen fibers. These results are also sustained by the study of Ng et al., which compared the degradation of intact collagen and hydrolyzed collagen from hydrogels and demonstrated that the hydrolyzed hydrogels show a much higher mass loss compared to the intact collagen hydrogel of around 20–25% throughout the 240 min, highlighting the important role of intact triple helical structure on the biodegradability of collagen hydrogels [37]. Other studies demonstrated that the microstructural properties, such as higher density combined with a lower porosity, play an important role in the degradation process and determine a slower degradation speed [38]. Also, in our study, results showed that the degradation was faster in hydrogels with higher porosity. Melero et al. tested five commercially available hyaluronic acid-based dermal fillers and showed similar enzymatic degradation profiles, each reaching approximately 60% hydrolysis within 48–72 h. The presence of unbound or short-chain hyaluronic acid, particle size, and molecular weight are anticipated to affect degradation kinetics [39,40]. While free or low-molecular-weight hyaluronic acid chains may continue to be extremely vulnerable to hydrolysis, smaller particles would provide more surface area for enzymatic attack [39,41]. Also, rheological compression and tensile tests would have provided valuable insight into the enzymatic degradation behavior of the hydrogels, but biomechanical tests were not performed in this study, representing a limitation, but also a perspective for future studies.

For the blood vessel model, AFSCs were differentiated in this study into EPCs and SPCs, as flow cytometry, molecular biology, and functional tests revealed. The blood vessel model was created by mixing the collagen–hyaluronic acid hydrogels with EPCs and SPCs differentiated from AFSCs. AFSCs are multipotent cells with plasticity to differentiate into many cell types under specific conditions, making them a good candidate for tissue engineering [42,43]. The stem cell ability to survive and differentiate is actively influenced by collagen, hyaluronic acid, and fibronectin [44]. The differentiation into EPCs was confirmed by the presence of a specific immunophenotype, expression of specific genes, the ability to form networks on Matrigel, and the capacity to incorporate LDL and *Ulex europeus* lectin. SPC differentiation was achieved after four weeks of cultivation in a specific medium supplemented with muscular growth factors, results confirmed by the specific morphology, gene expression pattern, and electrophysiological properties.

Using BIOCAD software v1.0, we printed a small-diameter 2-layer blood vessel model biocompatible with amniotic fluid stem cells. Our results showed a similar viability of cells between 72 and 80% after 7 days of culture and 78–88% after 21 days of culture for all three types of constructs. Using extrusion-based bioprinting, Ávila et al. created auricular constructs using a bioink made of human nasal chondrocytes, alginate, and nanofibrillated cellulose. Following bioprinting, cell viability was 70% and increased during a 28-day in vitro culture [45]. In another study by Müller et al., who extruded the alginate sulphate and nanocellulose bioink embedded with chondrocytes, the cell viability was kept at over 85% after 14 days of culture and 88% after 28 days [46].

Moreover, in this study, we demonstrated that hydrogels do not create oxidative stress for EPCs and SPCs, permit proliferation inside the vascular constructs, and are able to secrete nitric oxide and VEGF, which play an important role in vasodilation, adhesion, inflammation, control of smooth vascular muscle proliferation, activation of endothelial progenitor cells, and stimulation of angiogenesis and wound healing [47]. Our results showed that between 7 and 21 days, the nitric oxide concentration suffered minor modification that could be explained by its own production through both negative and positive feedback loops. Inhibiting nitric oxide synthase (NOS) activity and expression is how it often exerts negative feedback. However, under certain situations, especially in human cells, it can provide positive feedback to increase the expression of inducible NOS (iNOS) [44,47]. VEGF increases the activation of eNOS and production of NO required at the site of vessel formation by EPCs and regulates proliferation, apoptosis, and differentiation of EPCs [48].

Furthermore, immunohistochemical analysis showed that the constructs formed two distinct layers, without any mixing between the EPC and SPC hydrogels after 21 days of culture, aspects proved by the evidence of CD31 and CD 144 markers in the internal layer and smoothelin, and tropomyosin-1 markers in the external layer. These results suggest that these cells adhere to both the hydrogel and each other, respecting the structural model of a small blood vessel.

Thus, collagen–hyaluronic hydrogels are suitable biomaterials for creating vascular structures that can promote neovascularization and wound healing, facts also sustained by other studies. Duan et al. demonstrated that modifying the collagen hydrogel environment by increasing density, adding crosslinkers, or adding other polymers promotes the viability of vascular smooth muscle cells (VSMCs) derived from stem cells and increases proangiogenic activities through paracrine secretions [49]. Their research showed that VSMCs secreted more proangiogenic growth factors, such as VEGF, bFGF, ANG-1, PDGF, TGF-β1, SDF-1α, and MMP-2, as the density of the collagen 3D hydrogels increased and stimulated proliferation and migration of endothelial cells [49,50,51,52,53,54,55,56,57]. Dash et al. showed that the hydrated collagen scaffolds enhanced the secretion of proangiogenic factors bFGF, VEGF, and MMP-2 with an increase in collagen density but did not affect the secretion of anti-inflammatory cytokines and tissue remodeling growth factors such as IL-8, IL-10, SDF-1α, TGFβ, PDGF, Ang-1, and KGF [53]. Murphy et al. showed that fibrin hydrogel enhances the angiogenesis and anti-inflammatory ability of the encapsulated bone marrow stem cells (BMSCs), promotes the secretion of VEGF and prostaglandin E2, and accelerates wound healing in a three-dimensional endothelial human skin-equivalent model [54]. Furthermore, BMSCs in collagen hydrogels significantly promote wound healing, epithelial cell proliferation, re-epithelialization, and collagen deposition in the full-thickness wound of a mouse model. Hyaluronic acid hydrogel loaded with adipose mesenchymal stem cells increases wound angiogenesis and normal tissue remodeling [55]. Liang et al. showed that collagen–hyaluronic loaded with silver nanoclusters exhibits good porosity, mechanical characteristics, printability, and biocompatibility, all of which are advantageous for wound healing. Their findings also demonstrate that hydrogels encouraged fibroblast migration and proliferation in vitro as well as tissue regeneration and collagen deposition in vivo. These hydrogels produced a favorable wound repair effect and can be utilized as a functional biomaterial to support chronic diabetic wound repair [56,57].

## 4. Materials and Methods

### 4.1. Amniotic Fluid Stem Cell Differentiation and Characterization

The amniotic fluid cell cultures (AFSCs) were kindly provided by the Personal Genetics Diagnostics Laboratory upon informed consent of the patients in agreement with national and EU rules. This study was conducted in accordance with the Declaration of Helsinki and was approved by the Ethics Committee of Personal Genetics, No 952/15.03.2025. AFSCs were obtained by centrifugation of amniotic fluid at 1050 rpm for 10 min and the cell pellet was cultivated in AmnioMax medium (Thermo Fischer Scientific, Waltham, MA, USA), with medium change every 3 days.

#### 4.1.1. Cell Culture and Differentiation Conditions

After 10 days, the primary culture was passaged and cultured in differentiation-specific media supplemented with growth factors. Endothelial differentiation was done by culturing the AFSCs in M200 medium supplemented with 10% FBS (fetal bovine serum), 40 ng/mL of vascular endothelial growth factor (VEGF), 20 ng/mL of insulin growth factor (IGF-1), 10 ng/mL of epidermal growth factor (EGF), 10 ng/mL of basic fibroblast growth factor (bFGF), 100 μg/mL of penicillin, 100 μg/mL of streptomycin, and 50 μg/mL of neomycin (all purchased from Thermo Fischer Scientific, Waltham, MA, USA). Smooth muscle differentiation was done by culturing the AFSCs in M231 medium supplemented with basic FGF (2 ng/mL), EGF (0.5 ng/mL), heparin (5 ng/mL), IGF (2 µg/mL), and bovine serum albumin BSA (0.2 µg/mL) (Thermo Fischer Scientific, Waltham, MA, USA). The cells were maintained in these media for 4 weeks, and passaged when they reached subconfluence. Cell cultures were maintained at 37 °C with 5% CO_2_ and 21% O_2_ in a humidified atmosphere.

#### 4.1.2. Flow Cytometry Assay

Flow cytometry was used for assessing the expression of cell-specific surface markers (Gallios, Beckman-Coulter, Brea, CA, USA). AFSCs (1 × 10^5^ cells/marker) were stained with fluorochrome-conjugated (FITC—Fluorescein-isothiocyanate and PE—Phycoerythrin) primary antibodies against CD29 (integrin β1), CD31 (PECAM-1), CD49e (integrin α5), CD54 (ICAM-1), CD56 (NCAM), CD73, CD90 (Thy-1), CD105 (endoglin), CD146 (MCAM), and VEGFR2 (Beckman-Coulter). AFSCs were detached using trypsin (Sigma-Aldrich, St. Louis, MO, USA) and washed in phosphate-buffered saline solution (PBS). Cells were then incubated with the primary antibodies at room temperature in the dark for one hour. Further, the cells were washed and centrifuged at 400× *g*, 10 min, in PBS with 1% BSA. For negative controls, AFSCs were stained with the corresponding isotype-matched IgG antibodies (Beckman-Coulter, Brea, CA, USA). Flow cytometry data were analyzed using the Gallios software 1.0 (Beckman-Coulter, Brea, CA, USA).

#### 4.1.3. Gene Expression and Functional Characterization of EPCs

EPC gene expression levels were assessed by qRT-PCR. Total cellular RNA was isolated from cultured cells using RNeasy Mini Kit (Qiagen, Hilden, Germany), and reverse transcription reaction was performed using M-MLV polymerase, High-Capacity cDNA Reverse Transcription Kit (Thermo Fischer Scientific, USA). mRNA levels of endothelial-associated genes (PECAM-1 (CD31), ICAM-1, VWF, VE-Cadherin (CD144), and eNOS) were quantified using TaqMan hydrolysis probes (ThermoFischer Scientific, Waltham, MA, USA). The TaqMan assays are listed in Table S1 of Supporting Information. Quantitative Real-Time PCR reactions were carried out in a real-time thermocycler (ViiA7, Applied Biosystems, Foster City, CA, USA), following the manufacturer’s guidelines. The results were expressed using relative quantitation (2^−ΔCt^), where ΔCT represents the CT difference between values for AFSCs and EPCs.

Dil-Ac-LDL uptake assay: AFSC-derived EPCs were incubated with 6 µg/mL of Dil-AcLDL-PE (acetylated low-density lipoprotein conjugated PE, ThermoFischer Scientific, USA). Cells were incubated for 2 h at 37 °C with 5% CO_2_ and 21% O_2_, washed with PBS and fixed with 1% PFA (paraformaldehyde) for 10 min at room temperature.

Ulex europaeus lectin (UEA) binding capacity to EPCs: For the assessment of Ulex europaeus agglutinin (UEA) binding capacity to EPCs, cells were incubated with 10 µg/mL of FITC–Ulex europaeus lectin (Sigma-Aldrich, St. Louis, MO, USA) for 2 h, followed by a wash with PBS. The nuclei were counterstained with DAPI (1 mg/mL). The photomicrographs were taken with a digital camera, Digital Net Camera DN100, using an Eclipse TE300 microscope (Nikon, Tokyo, Japan).

Matrigel vascular tubes assay: To evaluate the formation of blood vessel networks in Matrigel, cells were seeded into 96-well plates at a density of 3.000 cells per well. Briefly, 50 μL of Matrigel (Sigma-Aldrich, St. Louis, MO, USA) was added to each well of 96-well plates and left to solidify for 30 min at 37 °C. After Matrigel polymerization, cell suspension was added and incubated for 4 h. Tube formation was observed using an Eclipse TE300 microscope (Nikon, Tokyo, Japan) equipped with a digital camera (Digital Net Camera DN100).

#### 4.1.4. Gene Expression and Electrophysiological Analysis of SPCs

The mRNA levels of muscle-associated genes (smoothelin, calponin-1, Myh11 (myosin heavy chain), α-tropomyosin, caldesmon-1) were quantified using TaqMan hydrolysis probes (ThermoFischer Scientific, Waltham, MA, USA). The assays are listed in Table S1 of the Supporting Information. In patch-clamp experiments, we used borosilicate glass capillaries (GC150F-10, Harvard Apparatus, Road Hollistone, MA, USA) pulled with PUL-100 equipment (WPI, Sarasota, FL, USA) and fire-polished with a home-made microforge to yield a resistance in solution between 2 and 3 MΩ. The patch-clamp setup included an inverted microscope placed on an antivibratory platform in a Faraday cage, a stage temperature controller (TC202A, Harvard Apparatus, Road Hollistone, MA, USA), and a resistive feedback amplifier (WPC-100, ESF electronic, Göttingen, Germany) connected to a Digidata 1322A AD/DA interface controlled by the pClamp8.2 software (Axon Instruments, Molecular Devices, Sunnyvale, CA, USA). The extracellular and pipette solutions, as well as the voltage-clamp protocols used, were similar to those from previously published studies [58].

Multiple voltage protocols were applied, including a general protocol for K^+^ cur-rents with depolarization steps from −60 to +60 mV with a duration of 300 ms from a holding potential of −80 mV; a double-voltage-ramp protocol (from −120 mV to +80 mV in 2 s and back to −120 mV at the same time, from a holding potential of −70 mV) (I NS double-ramp); standard protocol 2 of voltage clamp for the separation of the current of Ca^2+^ type L from the current of Na^+^ voltage-dependent. We applied the following pharmacological compounds: DIDS (4,4′-diisothiocyano-2,2′-stilbenedisulfonic acid) 100 µM, mibefradil 10 µM, and nifedipine 1 µM (Sigma-Aldrich, St. Louis, MO, USA).

### 4.2. Design, Bioprinting, and Characterization of Blood Vessel Constructs Using Collagen–Hyaluronic Acid Hydrogels and EPCs and SPCs

**Figure 8:**
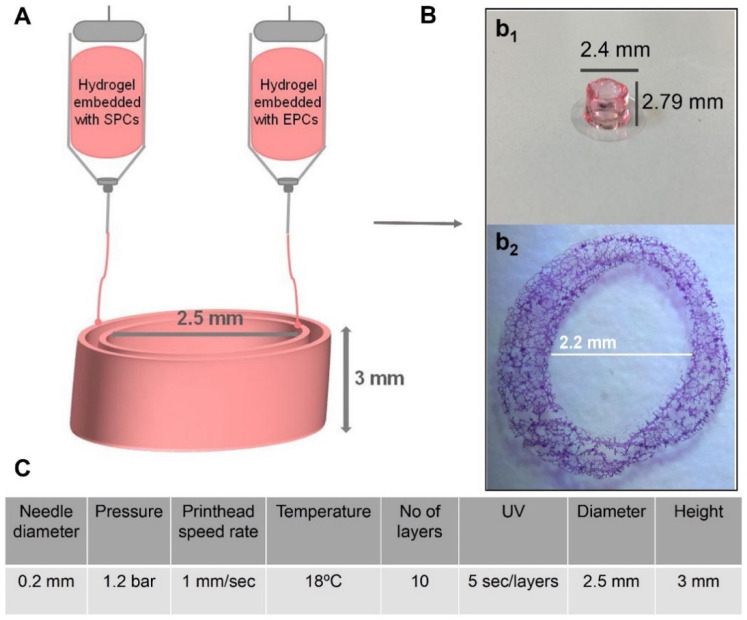
Schematic diagram of bioprinted vascular constructs (**A**) and optimal printing parameters of collagen–hyaluronic acid hydrogel-based vascular construct (**C**). Macroscopic morphology (**B**, **b1**) and H&E section of vascular constructs (**B**, **b2**).

A computer-aided design (CAD) software (BioCad, RegenHU, Villaz-St-Pierre, Switzerland) was employed to create the 3D models for bioprinting. A G-code series of instructions was to be used by the 3D bioprinter (3D Discovery, RegenHU, Switzerland). The 2 printheads (one with bioink encapsulating EPCs and the second with bioink encapsulating SPCs, respectively) moved according to G-code instructions, depositing bioink on slides. Bioprinting parameters were a 0.2 mm diameter needle, 1.2 bar extrusion pressure, 1 mm/s printhead speed rate, and 18 °C temperature. The diameter of vascular constructs was 2.5 mm, and the thickness and height of each layer were 0.3 mm. A 5 s polymerization followed each bioprinted layer. The number of cells was 10 million cells/1 mL of collagen–hyaluronic acid hydrogel. The amount was sufficient for printing 10 constructs. The schematic diagram in Figure 8 shows the bioprinting procedure.

#### 4.2.1. Hydrogel Preparation

Collagen hydrogel (H0) containing 2% peptides (collagen) was purchased from RegenHu (Villaz-St-Pierre, Switzerland). Using this hydrogel, we developed two other hydrogels (H2.5, H5) by adding hyaluronic acid (MW 1–1.6 MDa, 1%, Sigma-Aldrich, St. Louis, MO, USA) in 2 different proportions, 2.5% and 5%, respectively. According to the ratio, the ready-to-use collagen hydrogel (RegenHu, 2% collagen peptide/98% water, pH = 4.5) was mixed with hyaluronic acid powder and intensely stirred at 20 °C for 20 h. The pH was adjusted to 7.2–7.4 by the addition of 0.1–1N NaOH aqueous solution.

#### 4.2.2. Fourier Transform Infrared Spectroscopy (FTIR), Thermal Gravimetric Analysis (TGA), and Scanning Electron Microscopy (SEM)

FTIR spectra were recorded with a Nicolet iS50R spectrometer, at room temperature, in the measurement range of 4000–400 cm^−1^. Spectral collection was carried out in ATR mode at 4 cm^−1^ resolution. For each spectrum, 32 scans were co-added and converted to absorbance using OmincPicta software v9.0 (Thermo Scientific, Waltham, MA, USA). Thermal behavior of hydrogels was followed with an STA449C F3 system, TG-DSC (thermogravimetry–differential scanning calorimetry) from Netzsch (NETZSCH-Gerätebau GmbH, Selb, Germany), between 20 and 900 °C, in a dynamic (50 mL/min) air atmosphere. SEM imaging was obtained using the following protocol. The constructs were washed with PBS and fixed in 2.5% glutaraldehyde for 1 h at room temperature. Glutaraldehyde was then removed, and successive dehydration was carried out in ethanol (40%, 60%, 70%, 80%, 90%, and 100%) for 10 min each. SEM analysis was performed on a HITACHI S2600N electron microscope (Hitachi High-Technologies Corporation, Tokyo, Japan) at 25 keV on samples covered with a thin silver layer.

#### 4.2.3. Water Uptake

Water uptake was performed to demonstrate the hydrogel swelling behavior in the presence of PBS. Bioprinted constructs were weighed after printing, then immersed in PBS and weighed at 10 min, 30 min, 1 h, 2 h, 4 h, 8 h, and 24 h, respectively. The absorption rate was calculated according to the formula, where

(Ww − Wd)/Wd × 100(1)

Ww = construct weight after immersion in the fluid at time t; Wd = weight of dry scaffolds.

#### 4.2.4. Enzymatic Degradation

Enzymatic degradation of the vascular constructs was assessed using collagenase type I and hyaluronidase. Bioprinted vascular constructs were introduced into culture medium with collagenase type I (1 μg/mL) and hyaluronidase (10 μg/mL) (Merck, Rahway, NJ, USA) and then weighed at different intervals of time (10 min, 30 min, 1 h, 2 h, 4 h, and 8 h). Weight loss was calculated using the formula, where

(Wi − Wt) × 100/Wi(2)

Wi = initial weight of the construct; Wt = construct weight after time t

#### 4.2.5. Viability, Morphology, and Oxidative Stress Assays

Ten million EPCs and SMP per milliliter of bioink were mixed, and the behavior at 7 and 21 days after bioprinting was evaluated using LDH, GSH, and MTT assays. Cellular viability after 7 days and 21 days was evaluated using an LDH assay (Pierce™ LDH Cytotoxicity Assay Kit, ThermoFischer Scientific, Waltham, MA, USA). Lactate dehydrogenase (LDH) is a cytosolic enzyme present in many different cell types. Plasma membrane damage is followed by the release of LDH into the cell culture media. LDH can be quantified by a coupled enzymatic reaction in which LDH catalyzes the conversion of lactate to pyruvate via NAD+ reduction to NADH. Diaphorase then uses NADH to reduce a tetrazolium salt to a red formazan product that can be measured at 490 nm. Briefly, 50 µL of each sample medium was transferred to a 96-well plate and then 50 µL of the reaction mixture was added. The plate was incubated at room temperature for 30 min, protected from light. Then 50 µL of stop solution was added to each sample well and mixed by gentle tapping. The absorbance was measured at 490 nm and 680 nm using TECAN Infinite M200 (Männedorf, Switzerland).

Cellular stress was evaluated using the GSH assay (Glutathione Fluorescent Detection Kit, Thermo Fischer Scientific, Waltham, MA, USA). Glutathione (GSH) is an antioxidant found in eukaryotic cells. Reactive chemical species can cause a drop in GSH levels either by oxidation or reaction with the thiol group. A change in GSH levels is important for assessing stress response, potentially leading to apoptosis and cell death. The protocol followed the manufacturer’s specification, using a standard for absolute quantification. The fluorescent emission was read at 510 nm, with excitation at 390 nm using a TECAN Infinite M200 spectrophotometer (Männedorf, Switzerland).

The viability and proliferation were assessed using the MTT assay (Vybrant^®^ MTT Cell Proliferation Assay Kit, ThermoFischer Scientific, Waltham, MA, USA). The assay is a colorimetric method that allows quantitative assessment of proliferation, cell viability, and cytotoxicity. The viable cells reduce yellow tetrazolium salt MTT (3-(4,5-dimethylthiazol-2-yl)-2,5-diphenyltetrazolium bromide) to a dark blue formazan via mitochondrial enzymes. Briefly, the constructs cultivated for 7 days and 21 days, respectively, in 24-well plates, were incubated with 15 µL Solution I (MTT) at 37 °C for 4 h. Solution II (containing solubilization agents SDS and HCl) was added and pipetted vigorously to solubilize formazan crystals. After 1 h, the absorbance was read at 570 nm (TECAN Infinite M200, Männedorf, Switzerland).

#### 4.2.6. ELISA and Griess Assay

The secretion of VEGF from the endothelial progenitor cells at 7- and 21-day cultures in the construct was measured using the Human VEGF ELISA kit (Sigma-Aldrich, St. Louis, MO, USA) according to the manufacturer’s protocol. Nitric oxide (NO) was measured spectrophotometrically at 548 nm using the Griess Reagent Kit for Nitrite Determination (Molecular Probe, ThermoFischer Scientific, Waltham, MA, USA).

#### 4.2.7. Immunohistochemistry

Immunohistochemistry was performed on 5 μm thick cryosections of constructs at 7 and 21 days post-bioprinting. The sections were then incubated overnight, at 4 °C, with the following primary antibodies: monoclonal mouse anti-human CD31 (1:250), CD144 (5 μg/mL), smoothelin (1:100), and anti-human tropomyosin 1 (1:100) (Invitrogen, ThermoFischer Scientific, Waltham, MA, USA). The slides were washed three times with PBS for 10 min and then incubated for 2 h at room temperature, with the secondary antibodies coupled with streptavidin–biotin peroxidase, and diaminobenzidine (DAB) was used as the chromogenic substrate detection system. The pictures were taken using an inverted microscope with an incorporated digital camera system for imaging (Olympus CKX41 equipped with Sense XC30 camera, Shinjuku, Tokyo, Japan).

#### 4.2.8. Data Analysis

Data are expressed as mean ± standard deviation (SD) or mean ± standard error of the means as appropriate. In all instances, n signifies the number of replicates analyzed in a certain experimental condition. Statistical analysis was performed using Student’s *t* test or Fisher’s exact probability test, using a critical level of *p* < 0.05. For immunophenotyping and gene expression profiling, statistical analysis was performed using the one-way ANOVA method for correlated samples. The porosity of hydrogels was quantified using ImageJ software v1.54r. The immunohistochemistry-positive area quantification was performed using QuPath v0.7.0 software.

## 5. Conclusions

Since small-diameter vessels’ currently available synthetic grafts (<6 mm) have generally unsatisfactory results, this study attempted to create, using 3D bioprinting, a model of a small-diameter 2-layer blood vessel. Moreover, for the blood vessel model, collagen and hyaluronic acid hydrogels combined with endothelial and muscle progenitor cells derived from mesenchymal stem cells isolated from amniotic fluid were used. In addition to their great therapeutic potential, the use of amniotic fluid stem cells is ideal because they represent biological material that remains after prenatal diagnostic tests. Results obtained showed that collagen–hyaluronic acid hydrogels embedded with stem cells can be used for vascular constructs, meeting the desired requirements of biocompatibility and accuracy in reproducing the model created in the CAD software. Using 3D bioprinting, small-diameter 2-layer blood vessel models were created. All three vascular constructs presented good biocompatibility with amniotic fluid stem cells, with collagen hydrogel with 5% hyaluronic acid (H5) being the highest capacity of cell proliferation and secretion of angiogenic factors observed. Although the mechanical properties of the vascular constructs have not yet been investigated, the results are promising and could represent a starting point for further investigations. The next step will be translating in vitro results into an in vivo experimental model to demonstrate the stability and functionality of the blood vessel model within the complexity of a living organism.

## Data Availability

All data used to support the findings of this study are included within the article or in Supplementary Materials, and they are available upon request from the corresponding author.

## Supplementary Materials

The following supporting information can be downloaded at: https://www.mdpi.com/article/10.3390/ijms27094019/s1.
