# Supplementary material for: 3D Bioprinting of Blood Vessel Model for Improving Wound Healing
Source: Int J Mol Sci. 2026 Apr 30;27(9):4019. doi: 10.3390/ijms27094019 (PMC13164042; doi:10.3390/ijms27094019)
Supplement: Supplementary file 1 [file ijms-27-04019-s001.zip › ijms-4216929-supplementary.pdf]

| Gene | TaqMan assay code | Producer |
|---|---|---|
| CD31 (PECAM-1) | Hs01065279_m1 | Thermo Fischer Scientific, Waltham, Massachusetts, USA |
| eNOS | Hs01574665_m1 | Thermo Fischer Scientific, Waltham, Massachusetts, USA |
| CD144 (VE-cadherin) | Hs00901465_m1 | Thermo Fischer Scientific, Waltham, Massachusetts, USA |
| CD54 (ICAM-1) | Hs00164932_m1 | Thermo Fischer Scientific, Waltham, Massachusetts, USA |
| vWF (von Wilebrand Factor) | Hs01109446_m1 | Thermo Fischer Scientific, Waltham, Massachusetts, USA |
| Smoothelin | Hs01022255_g1 | Thermo Fischer Scientific, Waltham, Massachusetts, USA |
| Calponin 1 | Hs00959434_m1 | Thermo Fischer Scientific, Waltham, Massachusetts, USA |
| Myh11 (myosin heavy chain) | Hs00975796_m1 | Thermo Fischer Scientific, Waltham, Massachusetts, USA |
| α tropomyosin | Hs04398572_m1 | Thermo Fischer Scientific, Waltham, Massachusetts, USA |
| Caldesmon-1 | Hs00921987_m1 | Thermo Fischer Scientific, Waltham, Massachusetts, USA |
